# The clothes maketh the sign

**DOI:** 10.1007/s13244-016-0507-4

**Published:** 2016-06-07

**Authors:** Bryan Buckley, Victoria O. Chan, David P. Mitchell, Shaunagh McDermott, Ron L. Eisenberg, Eric J. Heffernan, Carole A. Ridge

**Affiliations:** Department of Radiology, Mater Misericordiae University Hospital, Dublin, Ireland; Department of Radiology, Massachusetts General Hospital, 55 Fruit St, Boston, MA 02114 USA; Department of Radiology, Beth Israel Deaconess Medical Center, 330 Brookline Avenue, Boston, MA 02215 USA; Department of Radiology, St Vincent’s University Hospital, Elm Park, Dublin, Ireland

**Keywords:** Pattern recognition, Visual, Radiography, Tomography, X-ray Computed, Magnetic resonance imaging, Ultrasonography

## Abstract

**Abstract:**

Pattern recognition is a key tool that enables radiologists to evoke certain diagnoses based on a radiologic appearance. In Shakespeare’s Hamlet, Polonius tells his son Laertes to dress well because “apparel oft proclaims the man”; this phrase is now expressed in modern parlance as “the clothes maketh the man”. Similarly in radiology, appearances are everything, and in the case of radiologic signs, occasionally “the clothes maketh the sign”. The radiologic signs described in this pictorial review resemble items of clothing, fabric types, headwear, or accessories and are found in the musculoskeletal, pulmonary, gastrointestinal, and genitourinary systems. These “clothing signs” serve as a useful visual trigger to help radiologists to identify particular disease entities.

***Teaching Points*:**

• *Pattern recognition enables radiologists to evoke a diagnosis based on radiologic appearance*.

• *The radiologic signs described in this review resemble clothing, fabric, or accessories*.

• *These “clothing signs” serve as visual triggers that evoke particular disease entities*.

## Introduction

In Shakespeare’s Hamlet, Polonius tells his son Laertes to dress well because “apparel oft proclaims the man” [1]; this phrase is now expressed in modern parlance as “the clothes maketh the man”. Similarly in radiology, appearances are everything, and in the case of radiologic signs, occasionally “the clothes maketh the sign”. These specific radiologic entities resemble clothing, fabric, headwear, accessories, and jewelry. These classic radiographic, computed tomographic (CT), sonographic, magnetic resonance imaging (MRI), and scintigraphic signs involve the musculoskeletal, pulmonary, gastrointestinal, and genitourinary systems and are described in this pictorial essay. These radiologic signs help radiologists recall their classic appearances and narrow a differential diagnosis.

## Musculoskeletal system

### Corduroy vertebra

**Fig. 1:**
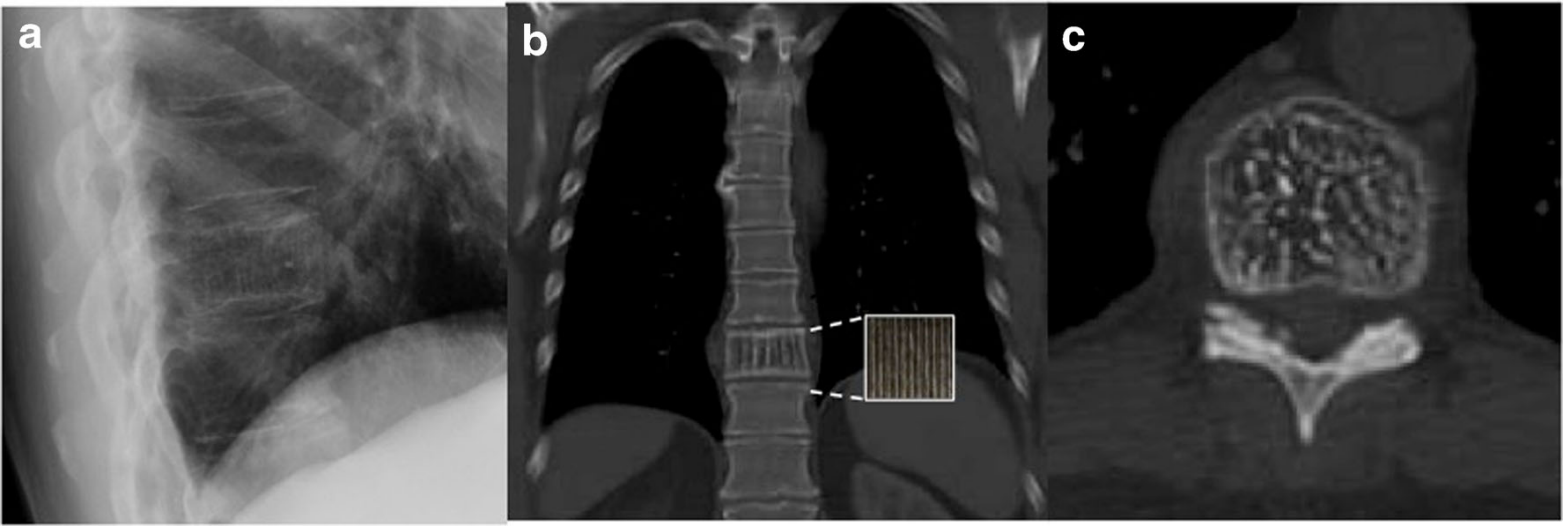
Coned image of a lateral thoracic spine radiograph (**a**) and corresponding coronal (**b**) and axial CT (**c**) images of an 80-year-old man. The images demonstrate low density interspersed by high-density vertical striations in the 10th thoracic vertebra similar to corduroy fabric (inset) consistent with a vertebral osseous hemangioma. There is a corresponding polka dot appearance on axial images, which represent the prominent trabeculae seen en face

The corduroy vertebra sign describes the appearance of thickened vertically oriented trabeculae seen in intraosseous hemangioma of the spine on lateral plain radiographs or sagittal CT of the spine (Fig. 1). The vertebral hemangioma is predominantly low in density interspersed by high density vertical striations similar in appearance to corduroy fabric [2]. This is due to the histopathologic structure of a hemangioma, which consists of thin-walled blood-filled vessels and sinuses lined with endothelium and interspersed with vertically oriented trabeculae of bone within fatty marrow [3]. On axial CT, a vertebral hemangioma exhibits a polka dot appearance due to the thickened trabeculae seen as small cross sectional areas of high attenuation surrounded by marrow fat (Fig. 1) [4].

### Inverted napoleon hat

**Fig. 2:**
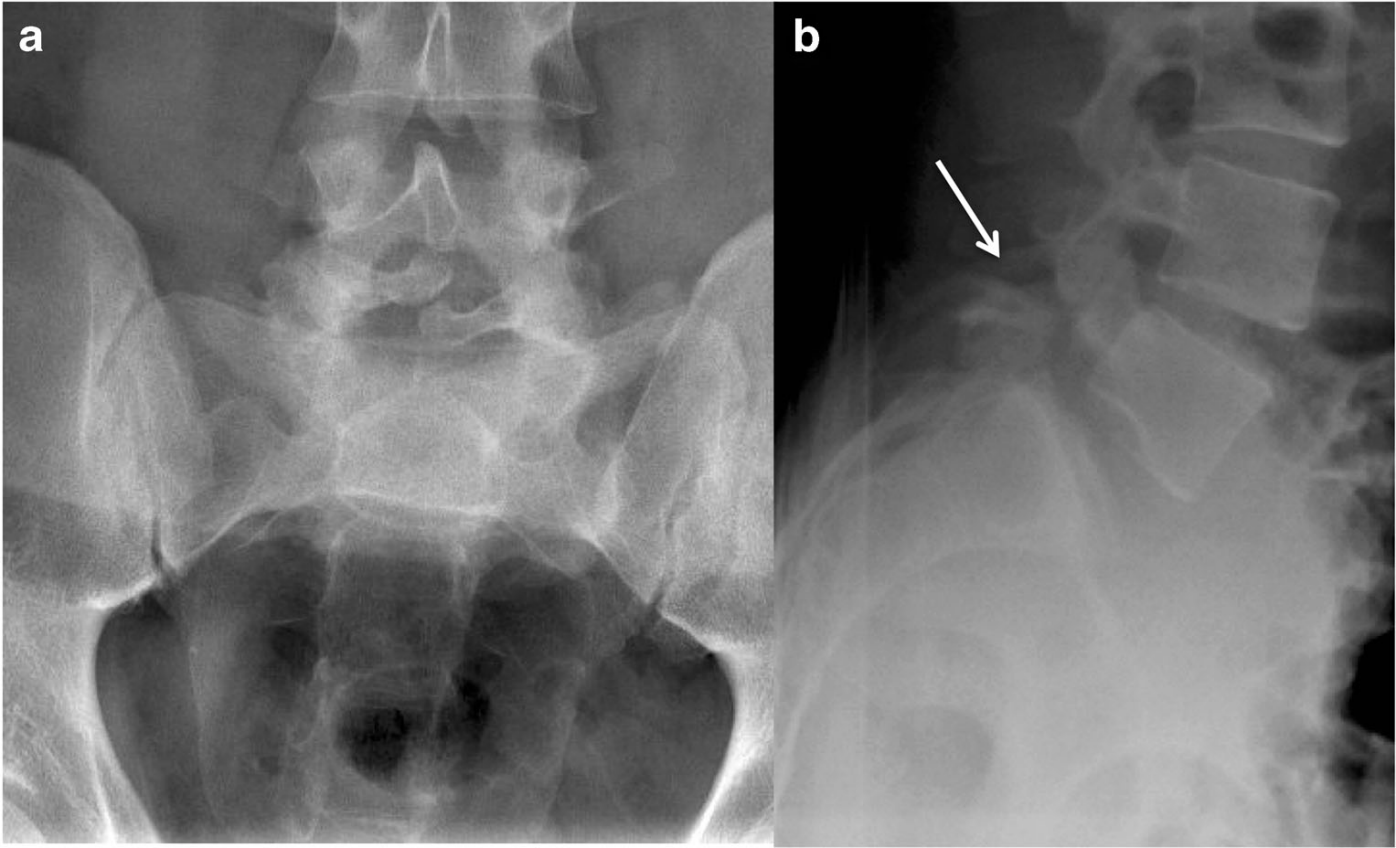
Frontal (**a**) and lateral (**b**) radiographs of the sacrum in a 35-year-old woman with lower back pain. The subluxed L5 vertebral body projected en face overlaps the first sacral vertebra and creates the appearance of an “inverted Napoleon hat” consistent with spondylolisthesis (**a**). The lateral projection confirms bilateral pars defects (**b**, arrow). The L5 transverse processes simulate the appearance of the bicorne hat made famous by Napoleon Bonaparte in the early nineteenth century

The inverted Napoleon hat sign refers to the appearance of the bicorne hat made famous by Napoleon Bonaparte in the early nineteenth century, which had a semi-circular fan-like appearance. Spondylolisthesis most commonly occurs at the lumbosacral junction, and in severe cases, the subluxed L5 vertebral body overlaps the sacrum; on the frontal view of a lumbosacral radiograph, the superimposition of L5 and the sacrum simulate the dome of the bicorne hat and the L5 transverse processes represent the hat’s tapered brim (Fig. 2) [5].

### Lace-like erosions

**Fig. 3:**
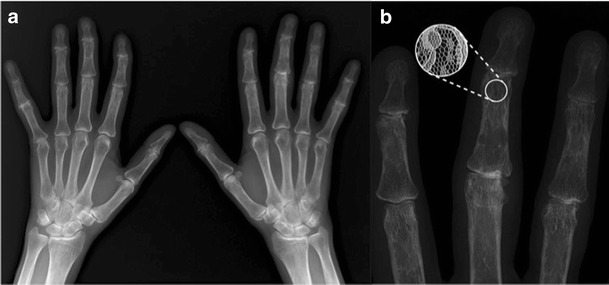
Bilateral hand radiographs and a coned image of the right second, third, and fourth phalanges in a 53-year-old patient with pulmonary sarcoidosis. Punched-out intramedullary cortical erosions resemble lace (**b**, inset) or latticework caused by chronic non-caseating granulomatous inflammation of the bone typically affecting the distal phalanges of hands and feet

Lace-like erosions are a radiologic manifestation of sarcoidosis caused by chronic noncaseating granulomatous inflammation of the synovium or bone, which typically affect the hands or feet. Granulomas result in punched-out cortical erosions or central lytic lesions within the medullary cavity. The characteristic appearance has been described as lacelike, latticework, or honeycombing (Fig. 3). The middle and distal phalanges are typical sites of involvement [6].

### Neck tie sternum

**Fig. 4:**
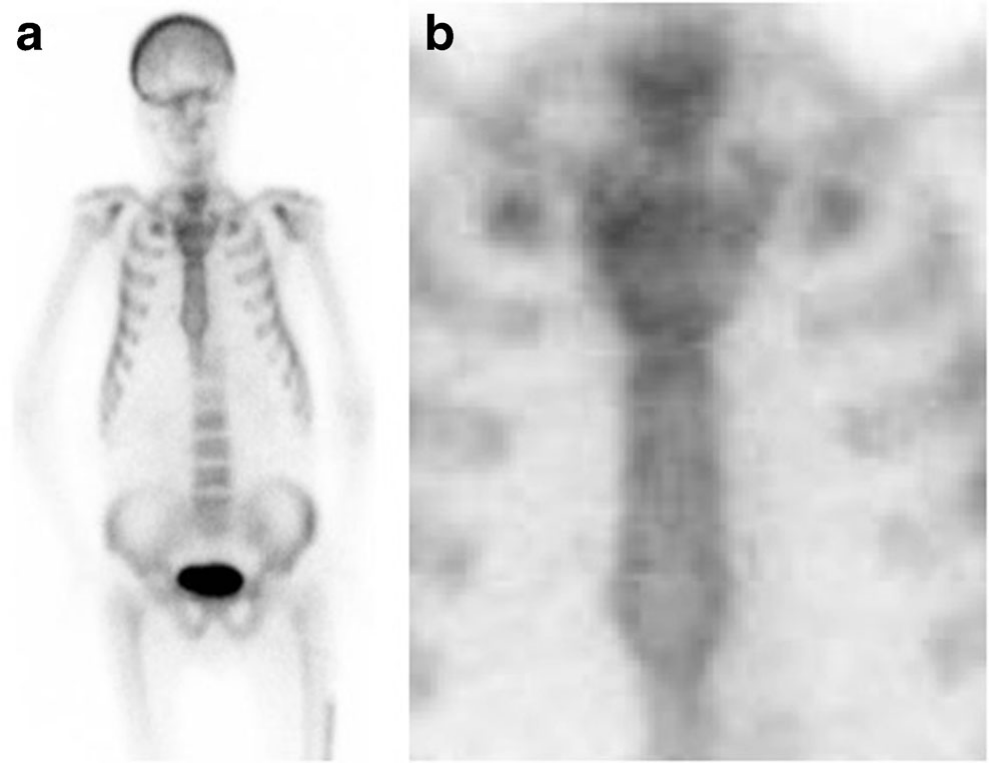
Bone scintigram in a 25-year-old man with renal failure and arthralgia. A frontal planar image (**a**) and magnified view (**b**) demonstrates expansion of the manubrium and sternum without manubriosternal joint expansion resulting in a necktie appearance. In addition, a prominent calvarium and faint visualization of the kidneys are also supportive of a diagnosis of renal osteodystrophy

Increased tracer uptake on bone scintigraphy within the sternum can give an appearance of a neck tie. This has been described most commonly in metabolic bone disease including renal osteodystrophy, hyperparathyroidism, and fluorosis [7, 8]. The neck tie sternum comprises expansion of the manubrium and sternal marrow without concurrent expansion of the manubriosternal joint. The latter results in a relatively narrow waist making the entire sternum appear like a neck tie (Fig. 4) [9]. The pathophysiologic basis for such an appearance is due to accelerated bone turnover and is usually accompanied by other features of metabolic bone disease including increased tracer uptake in the axial skeleton, long bones, and periarticular areas with prominent calvaria, faint visualization of the kidneys, and beading of the costochondral junctions [10].

### Rugger jersey spine

**Fig. 5:**
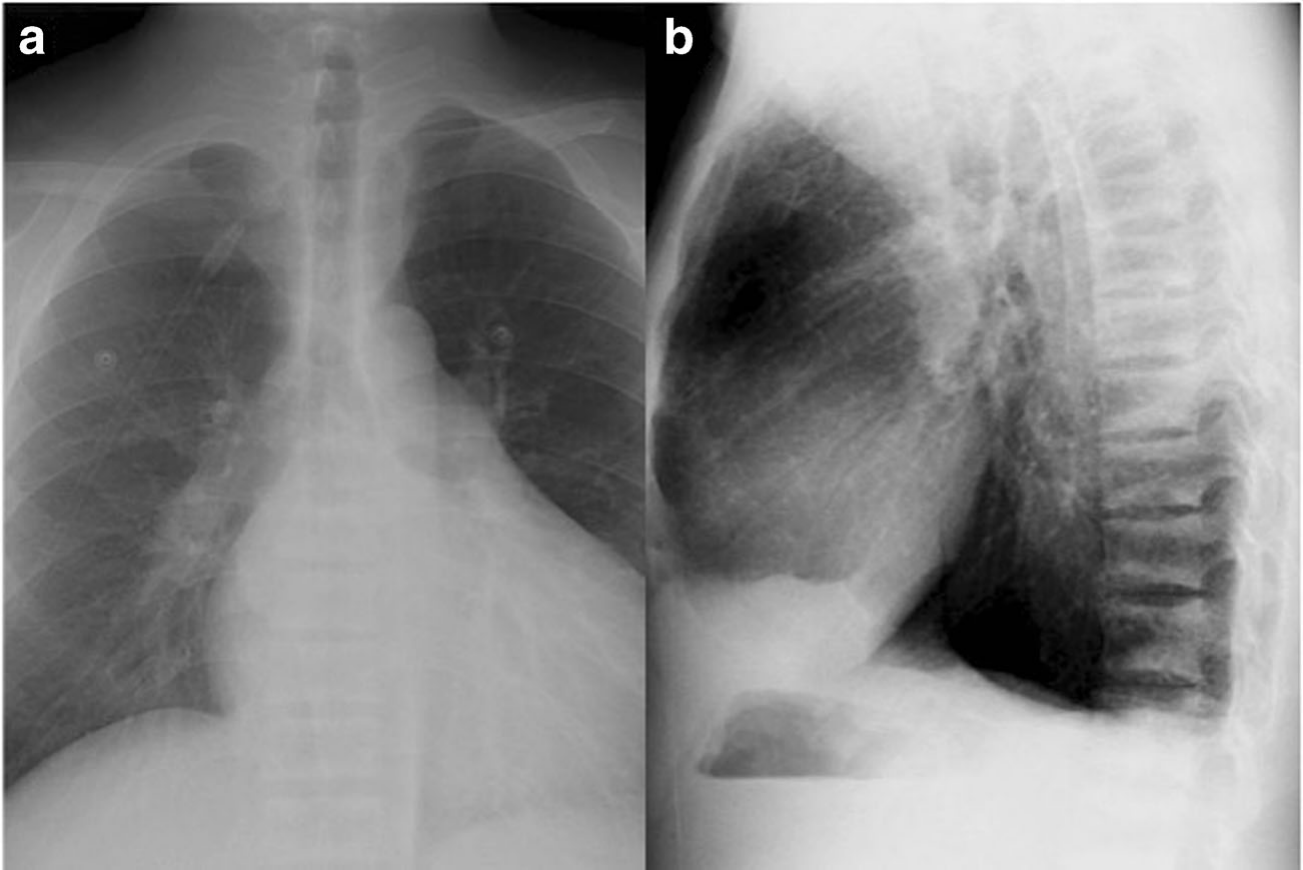
Frontal (**a**) and lateral (**b**) chest radiographs in a patient with renal failure. Sclerotic bands at the vertebral endplates with relative central lucency resemble the stripes on a rugby jersey. The sclerotic bands represent excess osteoid concentrated at the vertebral endplates. The chest radiograph also demonstrates enlargement of the cardiac silhouette due to a pericardial effusion (**a**) and a left pleural effusion on the lateral projection (**b**)

This sign is pathognomonic for osteosclerosis in the thoracic and lumbar vertebrae associated with secondary hyperparathyroidism of chronic renal failure demonstrated in 27 % of patients on radiographs [11]. Sclerotic bands, representing accumulations of excess osteoid, are seen along the superior and inferior endplates with a relative band of lucency in the centre of each vertebral body, giving alternating parallel bands analogous to the stripes present on an English rugby jersey (Fig. 5) [12]. The spinal canal and intervertebral disc spaces are normal.

### Absent bow tie sign of a bucket-handle tear

**Fig. 6:**
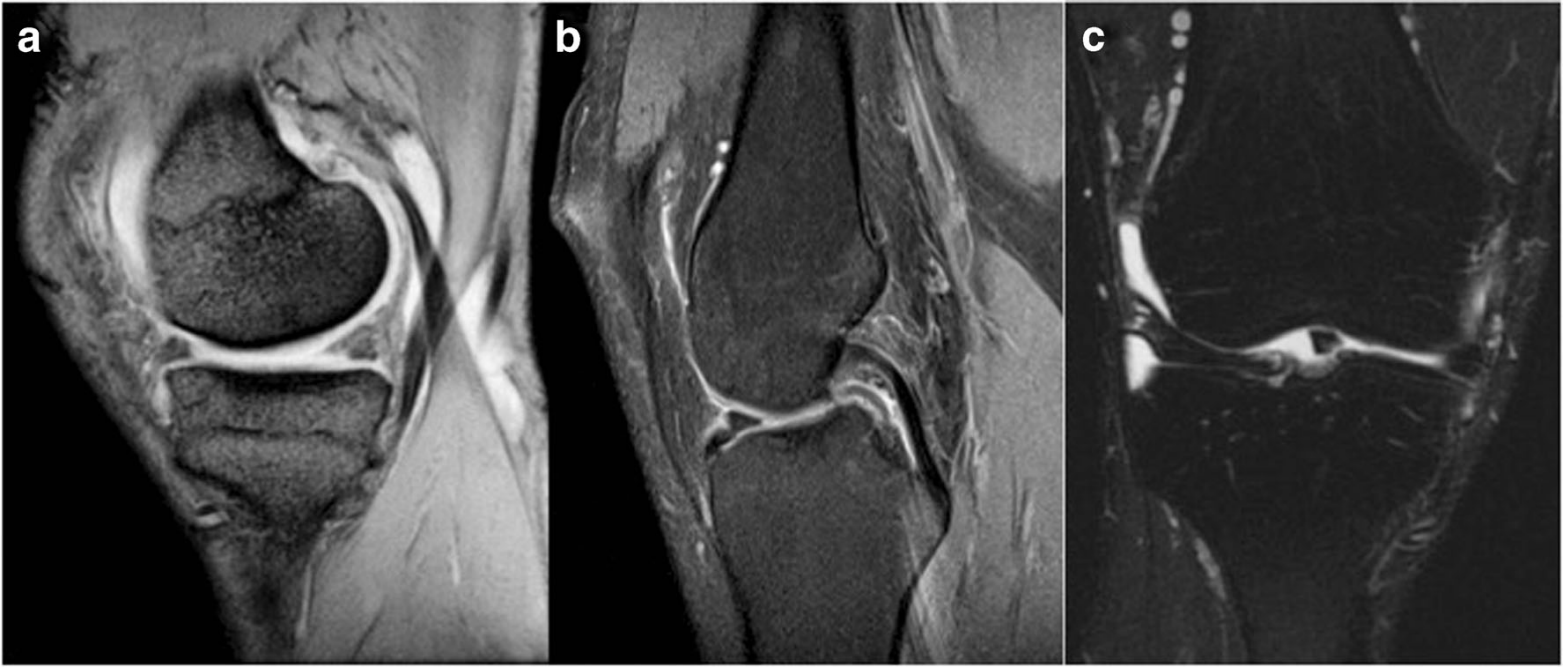
MRI of the left knee in an athlete with medial knee joint pain. Sagittal T2 weighted (**a**), proton density (**b**), and coronal short Tau inversion recovery (STIR) (**c**) sequences of the left knee demonstrate no body segment or “bow tie” joining the anterior to the posterior horns of the medial meniscus suggestive of a bucket-handle tear with confirmation being found in the displaced meniscus fragment in the intercondylar notch creating the “handle” of a bucket handle tear (**c**)

On sagittal MR images of the knee, a meniscus is considered normal when two consecutive images show the body of the meniscus in continuity with the anterior and posterior horns of the meniscus without evidence of a tear giving a “bow tie” appearance. When the sagittal images demonstrate only one or no body segments (“bow ties”), it is deemed positive for an absent bow tie sign and suggestive of a bucket-handle tear (Fig. 6) with confirmation being found in a displaced meniscus fragment elsewhere [13]. A bucket-handle tear, commonly involving the medial meniscus, typically consists of a vertical or oblique tear in the posterior horn that extends longitudinally through the body segment towards the anterior horn. The inner meniscal fragment is often displaced into the intercondylar notch creating the “handle”.

### Extra bow tie sign of a discoid meniscus

As the name suggests, a discoid meniscus is a disc-shaped meniscus (congenital variant) with the vast majority occurring on the lateral side of the knee. The discoid shape results in greater coverage of the tibia and is usually associated with increased thickness of the meniscus that may lead to abnormal shearing forces across the knee joint predisposing to meniscal tears. The presence of a discoid meniscus is suggested on MRI when three or more 5-mm-thick consecutive sagittal images demonstrate continuity of the meniscus between the anterior and posterior horns, producing an “extra bow tie” [14].

### Button sequestrum

**Fig. 7:**
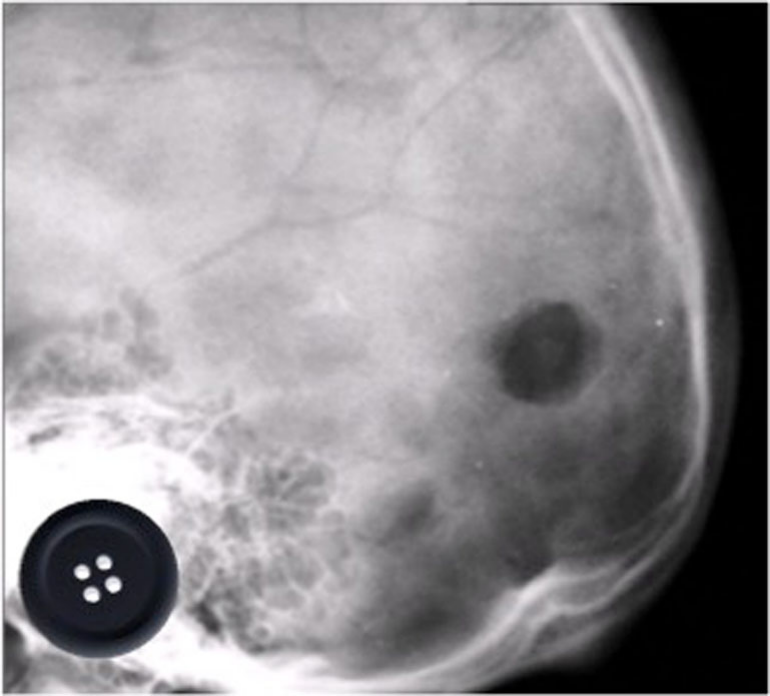
Lateral projection of the skull in a 63-year-old diabetic patient with fever and occipital scalp pain demonstrates a lucent lesion with a central opacity resembling a button (inset). The infectious organism destroys the bone, which is replaced by purulent material and granulation tissue, producing a lucency. Central opacity represents an island of dead bone (“button sequestrum”)

The classic button sequestrum sign is caused by a lucent lesion with a central ossific density (Fig. 7) and can be an uncommon manifestation of osteomyelitis, eosinophilic granuloma, fibrosarcoma, and lymphoma. In osteomyelitis, an infectious organism destroys the bone, which is then replaced by purulent material and granulation tissue, thereby producing the lucent area. The central opacity represents an island of dead bone and identification of such sequestrum can be an important indication for surgery in chronic osteomyelitis [15]. Initially described on radiographs, this sign can also be observed on CT scans.

### Bow tie sign of cervical spine facet dislocation

The most common orthopedic injury occurring after flexion-rotation trauma to the cervical spine is dislocation with unilateral locking of facets. Rotation of the cervical spine above the level of dislocation results in a diagnostic appearance on a true lateral radiograph. The articular facets of the vertebrae below the level of dislocation lie symmetrically parallel to each other so that only one set of superior and inferior articular facets per vertebra is visible. Above the level of dislocation, a double set of articular facets per vertebra will be present resulting in a “bow tie” appearance [16]. This is because of the rotation of the vertebrae, which now lie in an oblique position in relation to the X-ray beam.

### Bow tie appearance in vertebral compression

Vertebral compression fractures are the most common type of osteoporotic fracture and are diagnosed when >20 % of vertebral height is lost on imaging. In patients with severe vertebral compression fractures of the lumbar spine, the greatest loss of height of the vertebral body occurs in the center with relative sparing of the lateral aspects. This resembles a “bow tie” appearance in the coronal plane on imaging studies [17]. This morphology of vertebral compression fracture is specific to osteoporosis and is only seen in the lumbar spine due to weight distribution through the central body of the vertebrae.

## Neurologic system

### Ribbon ribs of neurofibromatosis type I

**Fig. 8:**
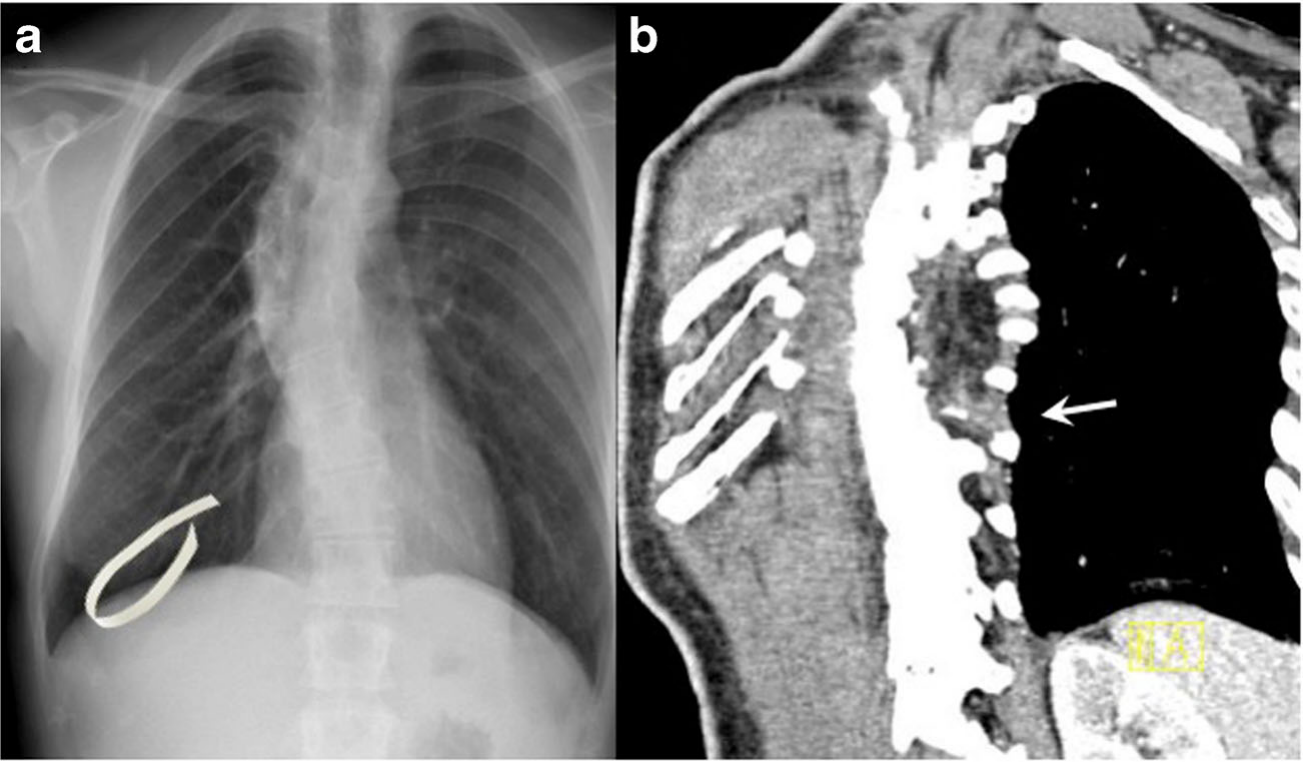
Chest radiograph (**a**) and coronal CT of the thorax (**b**) in a 25-year-old woman with cutaneous lesions demonstrating bilateral rib deformities that resemble ribbons. Well-defined erosions of either the superior or inferior margins of ribs secondary to plexiform neurofibromas and are a common manifestation of neurofibromatosis 1. The coronal CT reconstruction delineates a neurofibroma replacing the intercostal fat (**b**, arrow)

In the thorax, one of the most common skeletal manifestations of neurofibromatosis type I involve the ribs. Characteristic rib abnormalities include well-defined erosions of either the superior or inferior margins of one or more ribs with separation of adjacent ribs secondary to plexiform neurofibromas. This can result in marked deformity of the ribs due to either primary bony dysplastic changes or severe destruction which resembles “ribbons” on the chest radiograph (Fig. 8) known as the “ribbon ribs” deformity [18].

### Venus necklace sign in multiple sclerosis

**Fig. 9:**
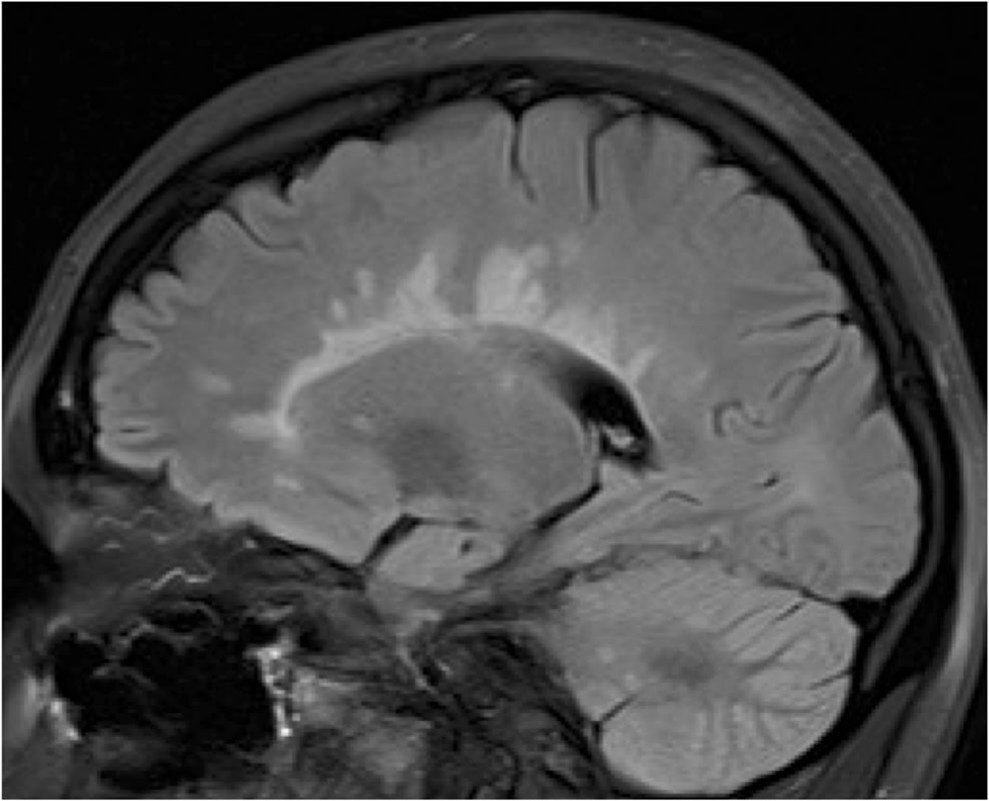
Sagittal fluid attenuated inversion recovery MRI sequence depicting several adjacent high signal pericallosal lesions resembling adjacent jewels in a Venus flytrap necklace, a style popular in the 1920s. The high signal lesions correlate with demyelinating plaques which typically arise in a perivenous location, the arrangement of lesions, at right angles to the corpus callosum, represents the distribution of callososeptal medullary veins, the appearance has also been described as “Dawson fingers”

Multiple sclerosis is a chronic relapsing disease, which is defined by symptoms and signs related to at least two sites of the central nervous system with a clinical course of relapse and remission. Magnetic resonance imaging (MRI) is sensitive for the detection of the responsible demyelinating plaques which frequently affect the corpus callosum (ref), amongst other sites. Multiple contiguous rounded T2 hyperintense lesions arranged at right angles to the corpus callosum or in the pericallosal deep white matter can manifest as the “Venus necklace sign” on sagittal T2 weighted or fluid attenuated inversion recovery MRI sequences. This appearance refers to the style of necklace made popular in the Art Deco period of the 1920s, which consisted of a necklace adorned by multiple rounded jewels in pronged settings, similar in appearance to an open Venus flytrap plant (Fig. 9). The arrangement of lesions represents the typical perivenous distribution of demyelinating plaques involving the callososeptal medullary veins, the appearance has also been described as “Dawson fingers”.

## Gastrointestinal system

### Beaded appearance of primary sclerosing cholangitis

**Fig. 10:**
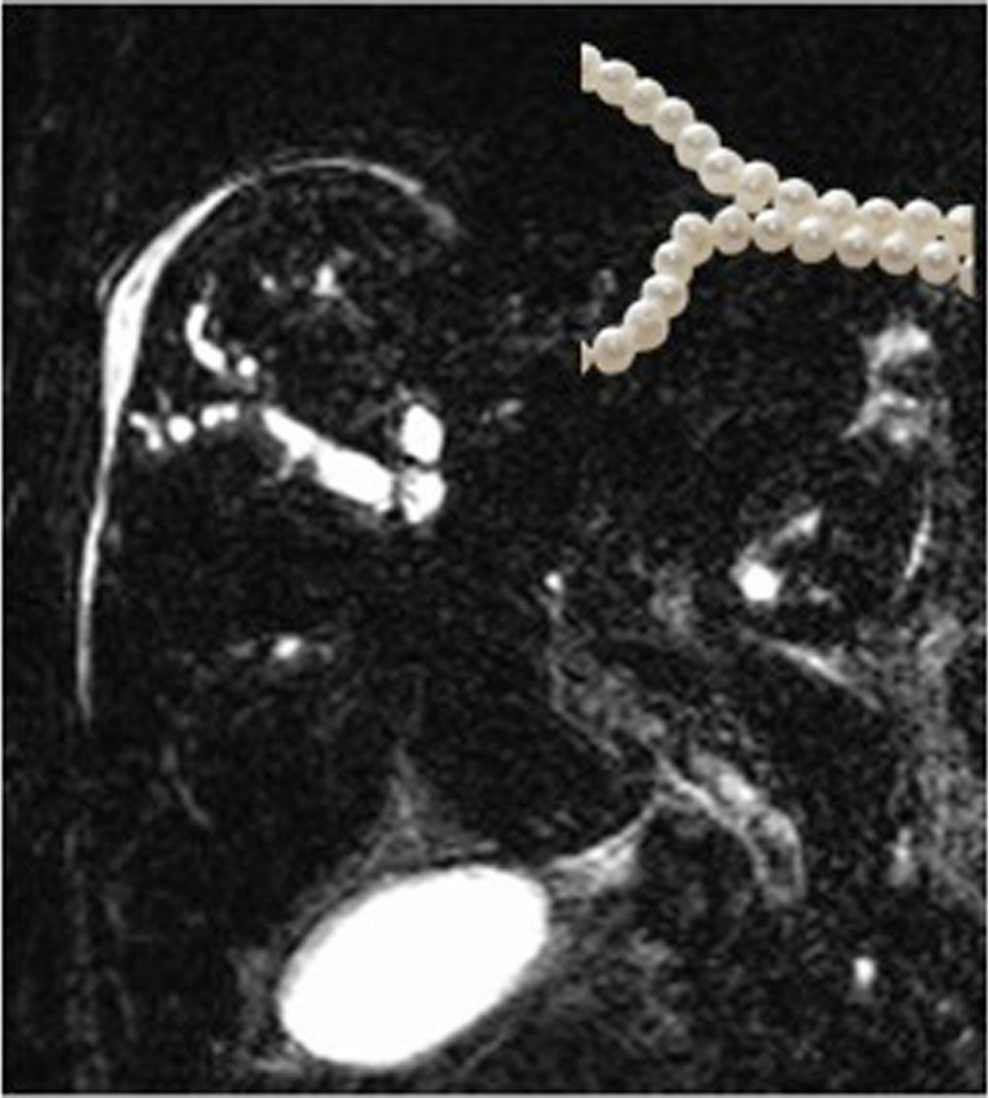
Magnetic resonance cholangiopancreatography (MRCP) in a 25-year-old man with jaundice and ulcerative colitis. Multifocal strictures involving the intrahepatic bile ducts produce a beaded appearance of the bile ducts (inset)

Primary sclerosing cholangitis is a chronic progressive disease of unknown etiology characterized by inflammation and fibrosis of the biliary tree. This causes diffuse stricture formation and eventually results in end-stage liver cirrhosis. Cholangiograms via endoscopic retrograde cholangiopancreatography (ERCP) or percutaneous transhepatic cholangiography (PTC) demonstrate multi-focal segmental strictures involving both the intra- and extrahepatic bile ducts. These can be diffusely distributed, short and annular, alternating with normal or slightly dilated segments to produce a “beaded” appearance (Fig. 10). With more advanced disease, long, confluent strictures are seen. In recent years magnetic resonance cholangiopancreatography has emerged as a less invasive alternative to ERCP/PTC and produce similar findings.

## String of pearls sign in small bowel obstruction

**Fig. 11:**
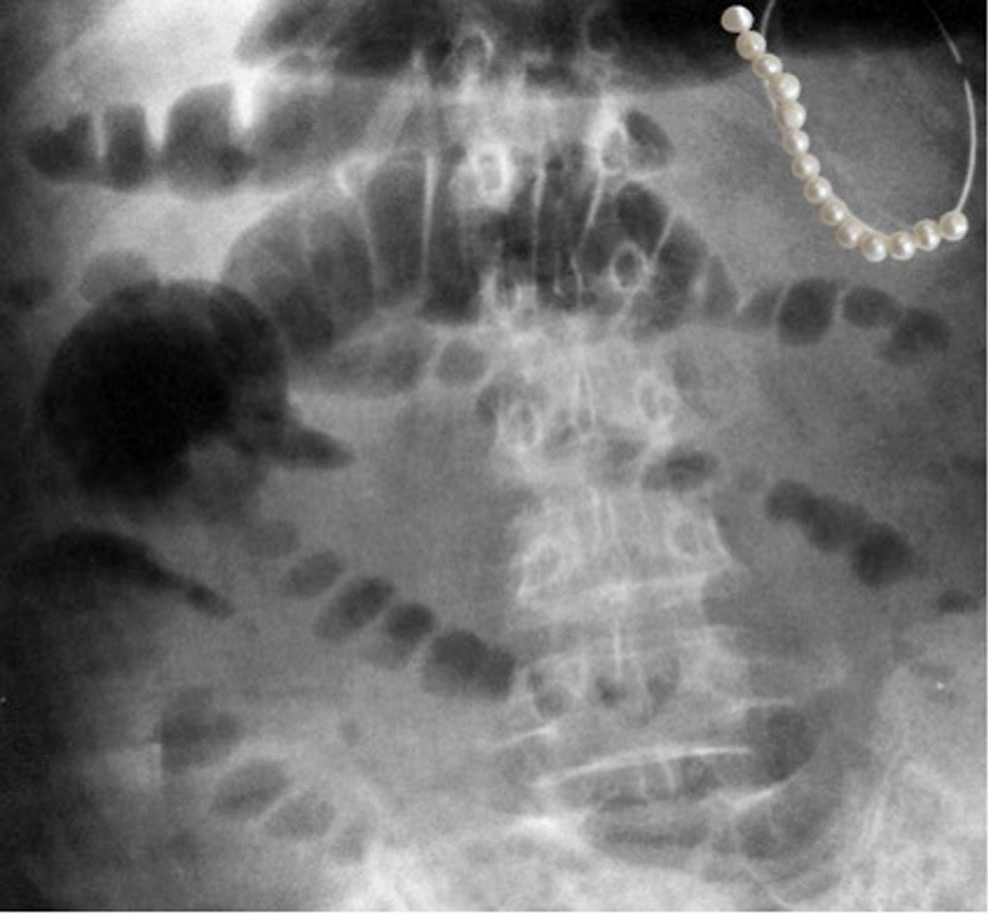
Plain film of the abdomen in a 52-year-old woman with abdominal pain and vomiting. Rows of air bubbles represent small amounts of air trapped between the valvulae conniventes of fluid-filled, dilated small bowel loops producing a “string of pearls” appearance (inset)

In some instances of small bowel obstruction, little or no air is present and the distended bowel loops are predominantly fluid filled. Thus, the supine abdominal radiographs may not demonstrate air distension of bowel. However, upright or decubitus radiographs may demonstrate air-fluid levels, or the "string of pearls sign". The obliquely oriented row of air bubbles represents small amounts of air trapped between the valvulae conniventes along the superior wall of the predominately fluid-filled, dilated small bowel loops. The meniscal effect of the surrounding fluid gives the trapped air an ovoid or rounded appearance – a “string of pearls” appearance (Fig. 11) [19].

## Pearl necklace sign in adenomyomatosis of the gallbladder

**Fig. 12:**
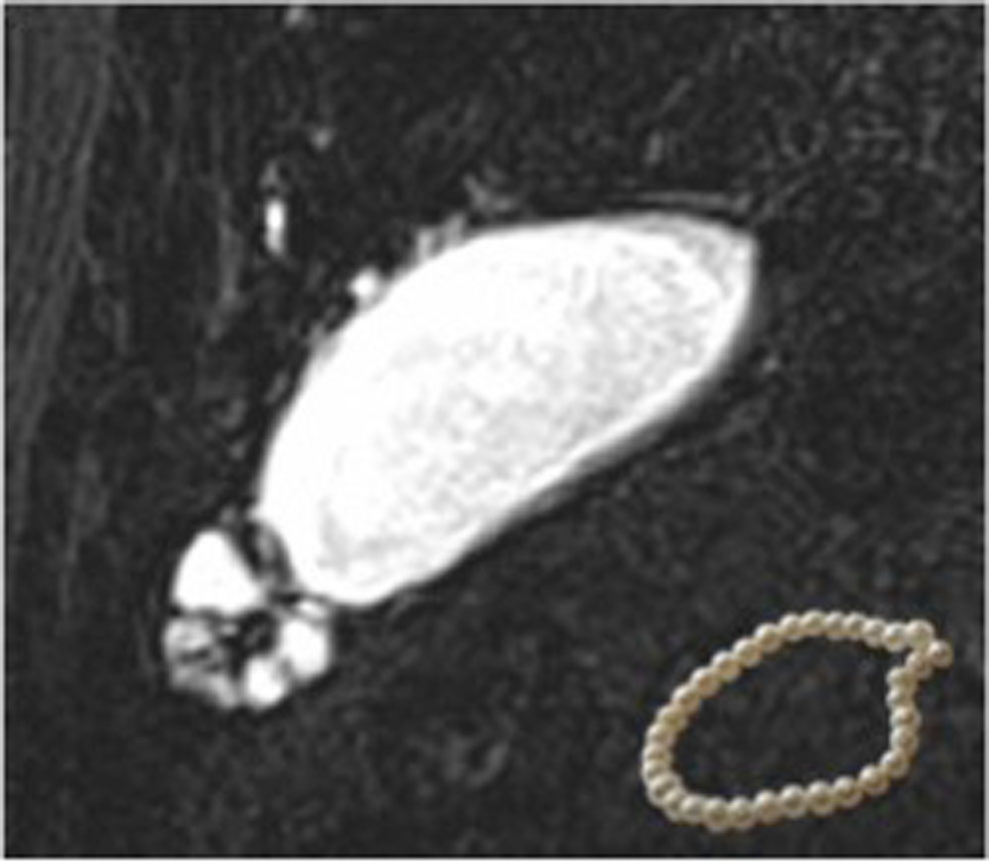
MRCP in a 69-year-old woman with abdominal pain. Outpouchings of mucosa in the muscularis of the gall bladder wall known as Rokitansky-Aschoff sinuses in the gall bladder fundus produce the “pearl necklace sign” (inset). This is likely to be an incidental finding

Pathologically, adenomyomatosis of the gallbladder is defined as epithelial proliferation and hypertrophy of the muscularis of the gallbladder, with outpouchings of the mucosa into the thickened muscular layer known as Rokitansky-Aschoff sinuses. It is a relatively common disease found in 2–5 % of specimens obtained at cholecystectomy. The “pearl necklace sign” indicates the presence of Rokitansky-Aschoff sinuses within the thickened gallbladder wall on MRCP (Fig. 12) [20].

### Phrygian cap

**Fig. 13:**
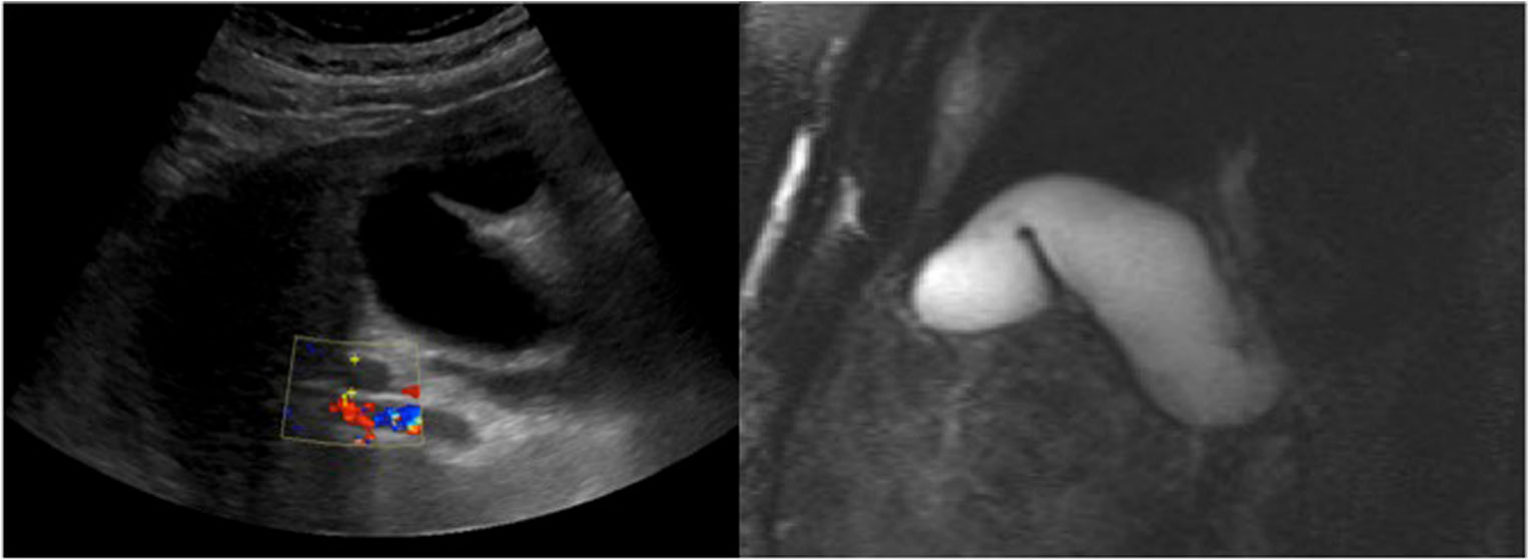
Transverse ultrasound and half Fourier acquisition single shot turbo spin echo (HASTE) maximal intensity projection (MIP) image in the coronal plane of a 44-year-old patient with abdominal pain. The gall bladder was normal and images demonstrated a normal variant whereby the gallbladder fundus is folded giving a “Phrygian cap” appearance

The Phyrygian cap is a common normal variant of the gall bladder which occurs when there is folding of the gall bladder fundus upon itself. It resembles a hat worn by the inhabitants of ancient Phrygia circa 1200 B.C. It is a common incidental finding on ultrasound, CT, and MRI imaging of the gallbladder and produces no symptoms (Fig. 13).

### Comb sign in Crohn's disease

**Fig. 14:**
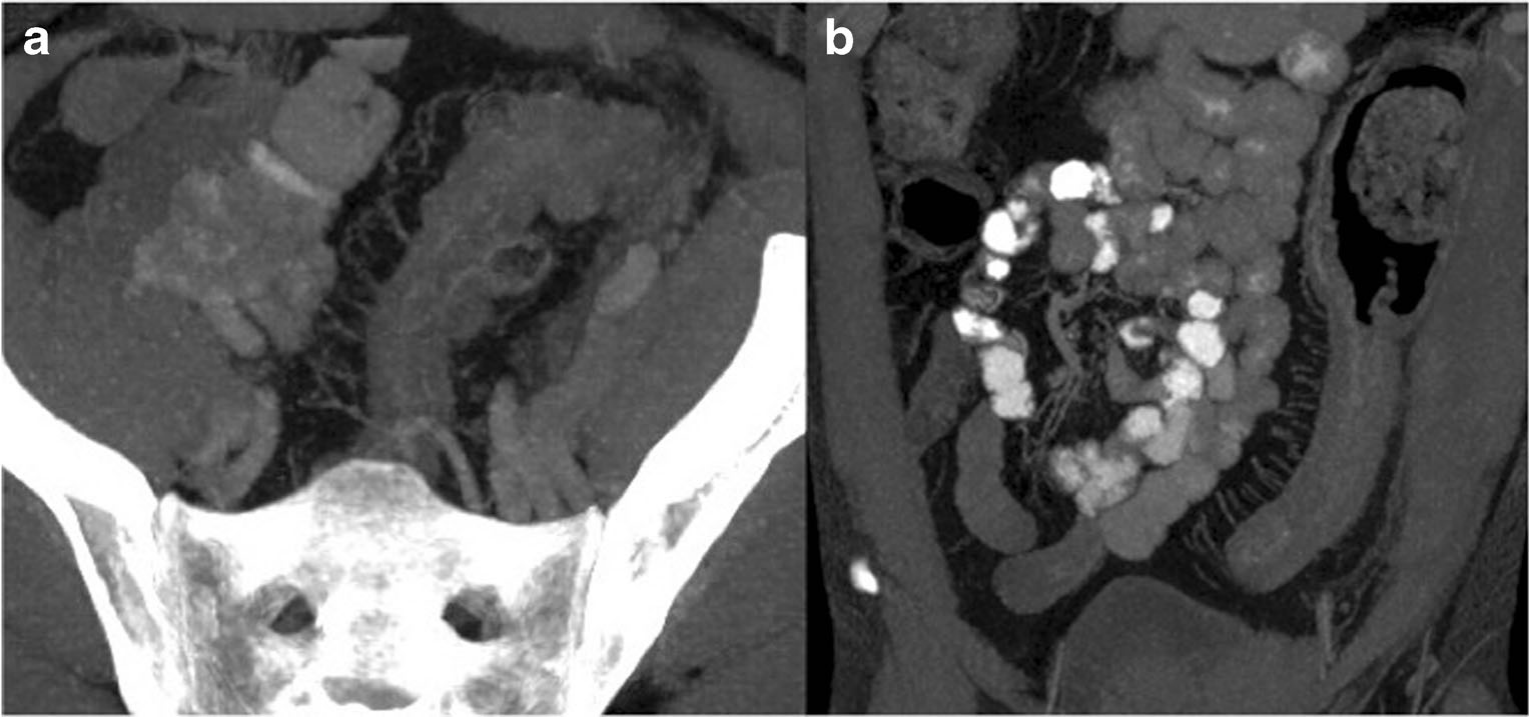
Axial and coronal thick MIP images of a 30-year-old man with an acute flare of Crohn's disease. Interposed fibrofatty proliferation and vascular distension produces a striated appearance resembling teeth in a comb and are indicative of active Crohn's disease

Crohn's disease is a chronic granulomatous inflammatory disease of the gastrointestinal tract with a tendency toward remission and relapse. The comb sign consists of interposed mesenteric fibrofatty proliferation and vascular distension, which gives the appearance of teeth of a comb. The sign is associated with active Crohn's disease and has been shown to correlate well with serum inflammatory markers (Fig. 14).

## Genitourinary system

### String of beads appearance in renal artery fibromuscular dysplasia

**Fig. 15:**
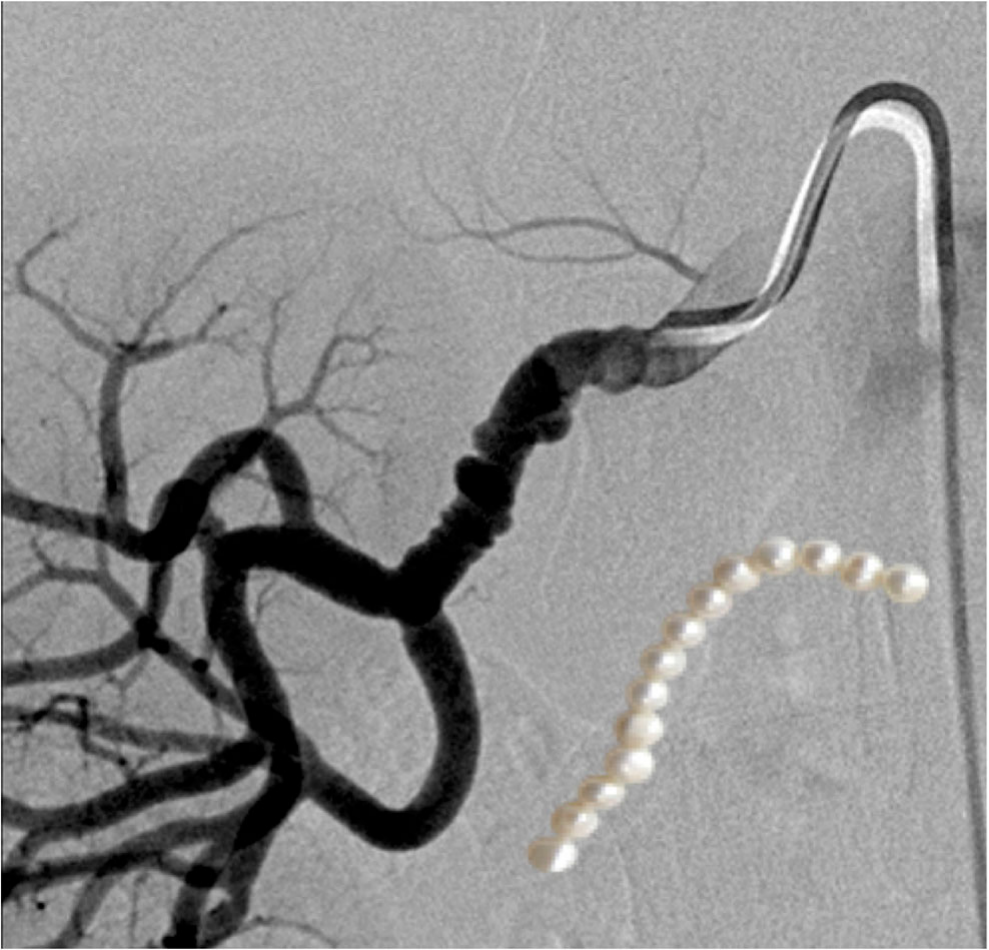
Digital subtraction angiogram in a 35-year-old woman with uncontrolled hypertension demonstrates contiguous relative stenoses alternating with fusiform aneurysmal dilatation of the right renal artery due to fibromuscular dysplasia resembling a string of beads (inset). Fibromuscular dysplasia is characterized by fibrous or muscular hyperplasia in one or more layers of the renal artery wall, producing this appearance

Fibromuscular dysplasia is a slowly progressive disease attributed to be the most common cause of renovascular hypertension in young and middle-aged women due to renal artery stenosis. The lesions characteristically affect the distal two thirds of the renal artery and are usually multi-focal with alternating zones of stenosis and aneurysms. This gives the classic “string of beads” appearance on angiograms (Fig. 15). Digital subtraction angiography is the gold standard for diagnosis, but in recent years, the renal arteries have also been evaluated by non-invasive means with CT angiography (CTA) or MR angiography (MRA). An advantage of CTA is that both the wall and lumen of the pathologic vessel wall can be visualized. MRA also produces excellent contrast-enhanced angiograms without the use of iodinated contrast.

## Signet ring sign in renal papillary necrosis

**Fig. 16:**
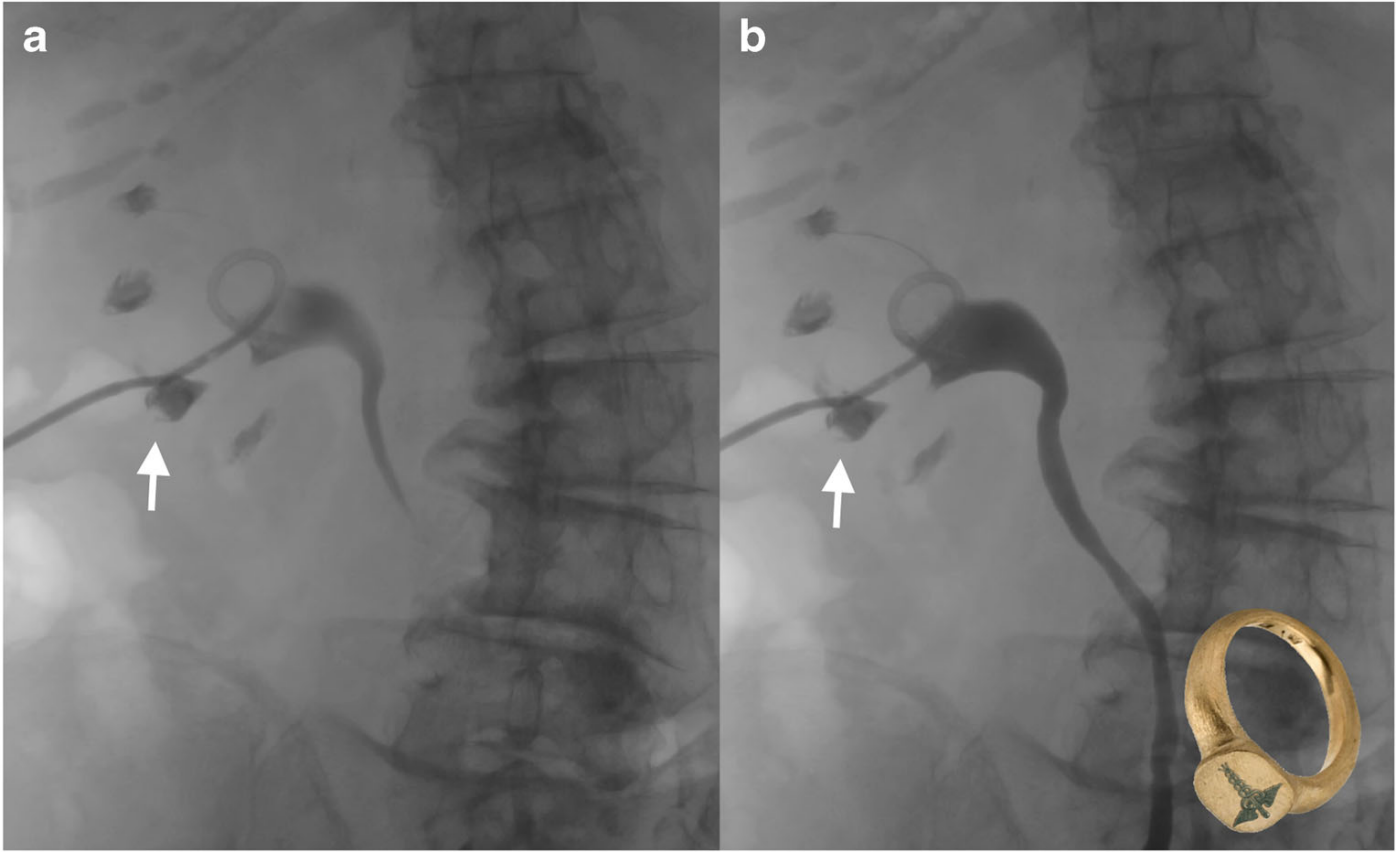
Antegrade pyelogram in an 85-year-old woman with acute on chronic renal failure and sepsis requiring nephrostomy placement. Antegrade pyelographic images demonstrate opacified renal calyces, a filling defect in the centre of a lower pole calyx (**a**), which persists on a delayed image (**b**) is consistent with a sloughed papilla as a result of necrosis (arrow), the filling defect is thought to resemble the jewel or insignia of a signet ring

Renal papillary necrosis is not a pathologic entity, but rather a descriptive term for necrosis of the renal papillae. The renal medulla and papillae are vulnerable to ischemic necrosis because of the peculiar arrangement of their blood supply. The “signet ring” sign is due to the necrotic papillary tip remaining within the excavated calyx when the calyx is filled with contrast material and resembles a signet ring, whereby the jewel or insignia represents the sloughed papilla outlined by contrast (Fig. 16) [21].

## Lace-like appearance of a hemorrhagic ovarian cyst

**Fig. 17:**
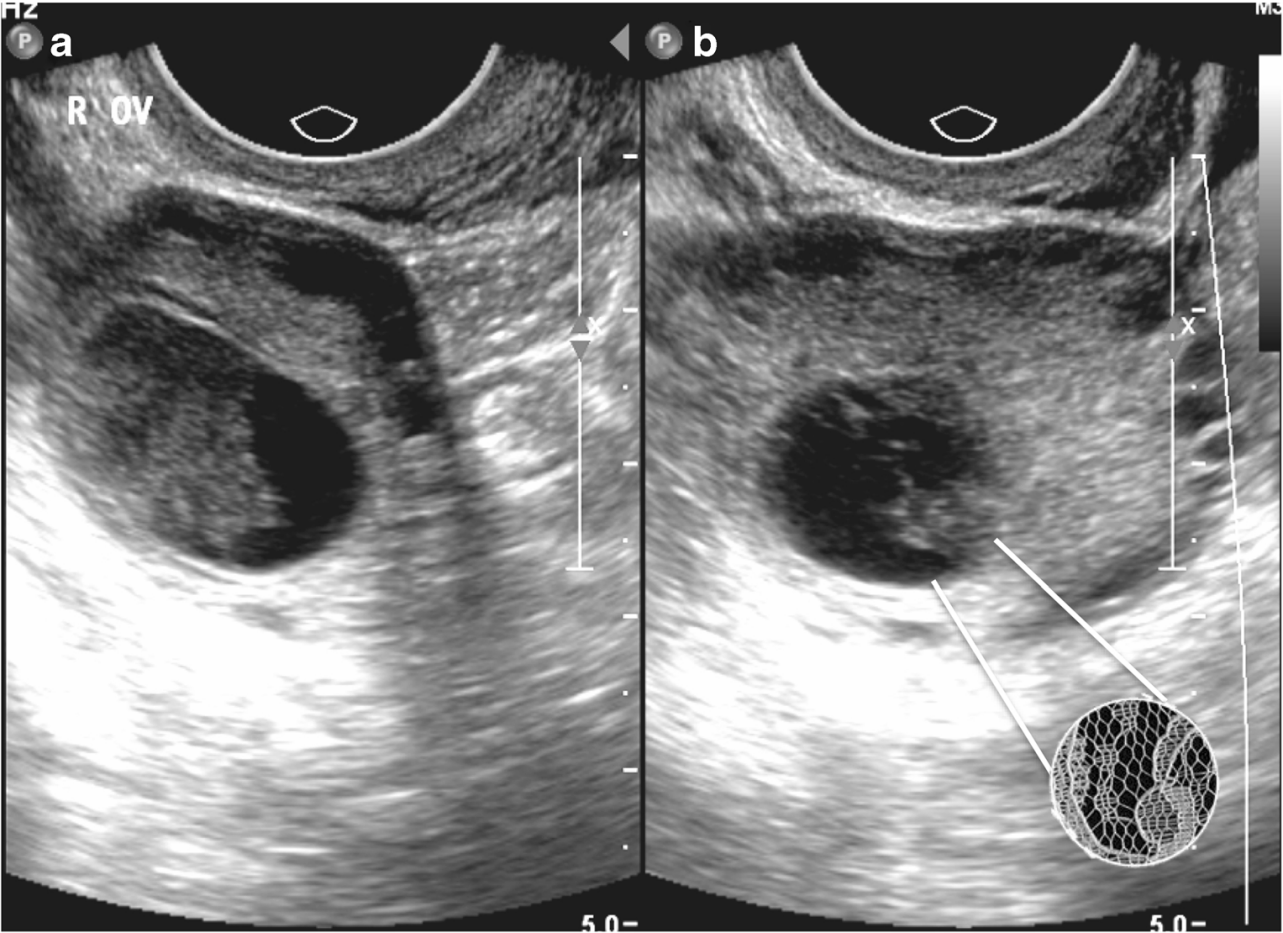
Endovaginal ultrasound in a 21-year-old woman with right iliac fossa pain. Transverse and longitudinal images (**a**, **b**) of the right ovary demonstrate fine interdigitating septations within an ovarian follicle, which give a lace-like or reticular appearance. The cystic mass can have posterior enhanced throughtransmission as in this case

Hemorrhage within an ovarian cyst is represented sonographically by an adnexal mass with fine interdigitating septations which give a lace-like or reticular appearance. The cystic mass can have posterior enhanced through-transmission and absence of color Doppler flow within the fine septations, which in fact represent fibrin strands (Fig. 17) [22].

## String-of-pearls appearance of polycystic ovarian syndrome

**Fig. 18:**
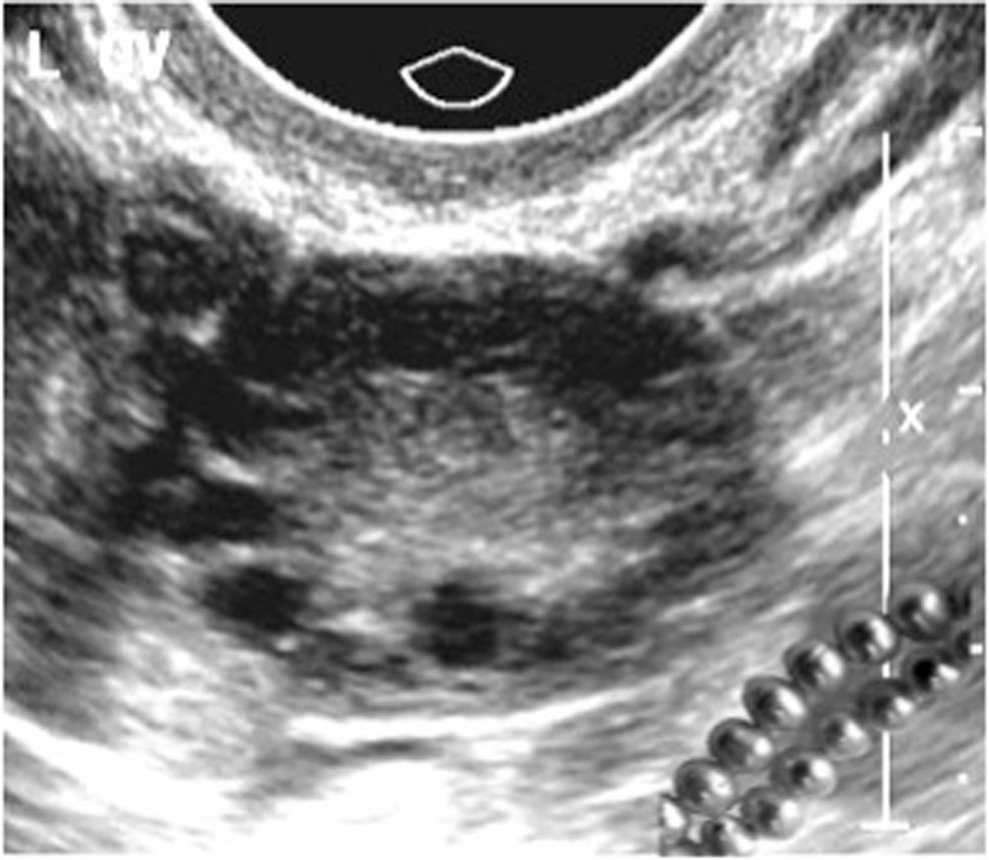
Endovaginal ultrasound images in a 26-year-old woman with hirsuitism and elevated body mass index. Numerous small cysts line up on the periphery of the ovary producing a “string-of-pearls” appearance (inset). The small cysts are arranged in a subcapsular distribution around an echodense ovarian stroma

The string of pearls sign can be used to diagnose polycystic ovary syndrome (PCOS) on ultrasound. It refers to the appearance of the ovary when numerous small cysts line up on the periphery of the ovary in a “string-of-pearls” appearance (Fig. 18). Ultrasonographic criteria for establishing the diagnosis of PCOS include 25 or more cysts that are 2–8 mm in diameter arranged in a subcapsular distribution around an echodense stroma [23].

## Cardiopulmonary system

### Boot-shaped heart

**Fig. 19:**
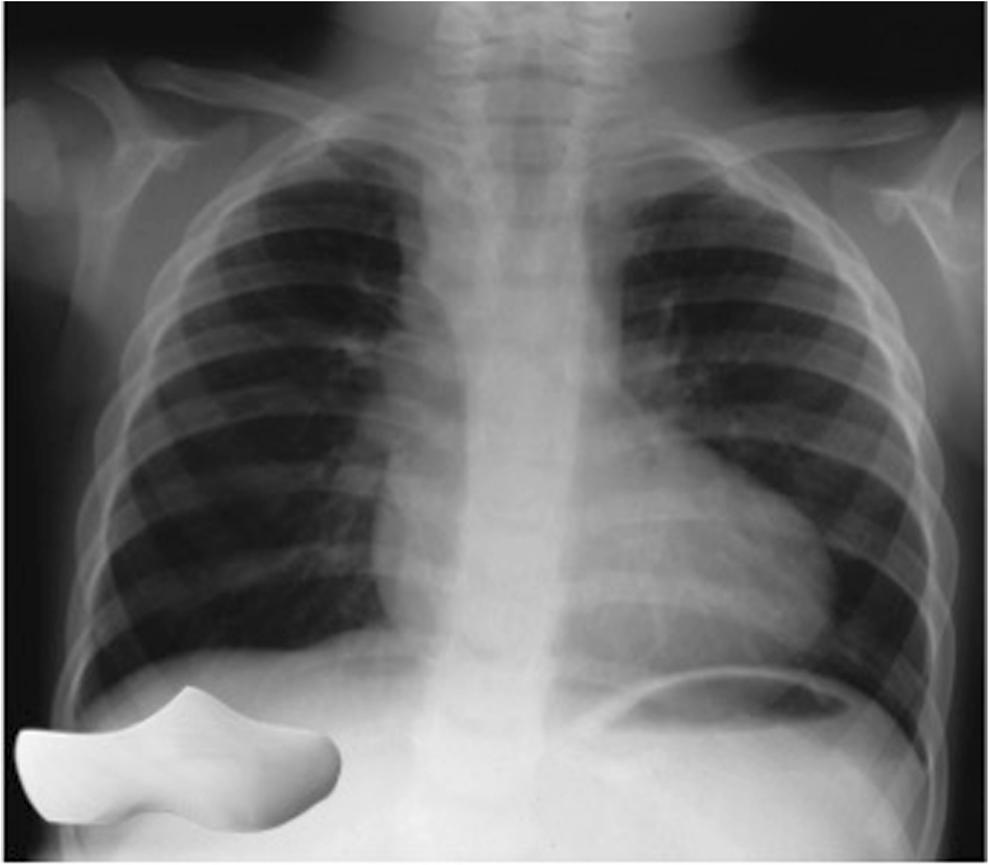
Chest radiograph in a 22-year-old man with corrected tetralogy of Fallot. The cardiac silhouette resembles a boot or a “sabot”, a shoe once worn by French peasants (inset), the large “toe” of the boot is caused by right ventricular hypertrophy and the narrow “ankle” of the boot is due to a small main pulmonary artery

The boot-shaped heart sign is a radiographic finding in patients with tetralogy of Fallot which consists of obstruction of the right ventricular outflow tract, ventricular septal defect (VSD), overriding of the aorta above the VSD, and right ventricular hypertrophy [24]. The toe of the boot is formed by the upward pointing cardiac apex caused by right ventricular hypertrophy, while the narrow ankle of the boot results from a hypoplastic or absent main pulmonary artery (Fig. 19). The sign is also referred to using the French term “coeur en sabot”, which refers to the traditional shoe made of a single piece of wood worn by farmers and workers in the Netherlands and France in the eighteenth and nineteenth centuries (Fig. 19).

## Finger in glove

**Fig. 20:**
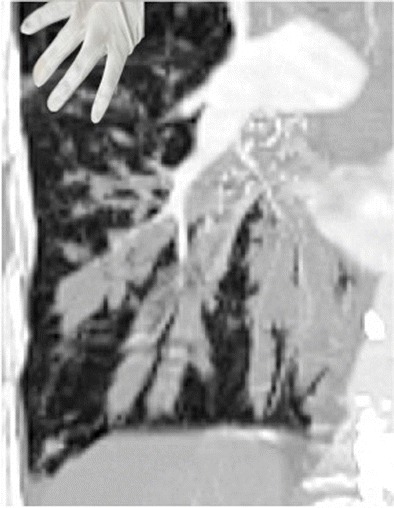
Coronal reconstructed CT in a 63-year-old woman with a chronic cough due to bronchiectasis and mycobacterium avium complex infection. CT demonstrates branching dilated airways filled with inspissated mucus resemble fingers in a glove (inset)

Bronchiectic airways filled with respiratory secretions resemble “fingers in a glove”, the branching dilated airways give the appearance of fingers and the inspired mucus comprises the radiodense fingers in the glove. The radiologic sign is classically associated with allergic bronchopulmonary aspergillosis (ABPA), a condition that arises most commonly when a patient with asthma develops superinfection with Aspergillus fumigatus and bronchiectasis [25]. It can, however, occur in any obstructive (e.g. bronchial tumours, congenital atresia) or non-obstructive (e.g. cystic fibrosis) form of bronchiectasis where there is inspissation of secretions in the dilated bronchi, as in Fig. 20, in a patient with chronic mycobacterium avium complex infection.

## Signet ring sign of bronchiectasis

**Fig. 21:**
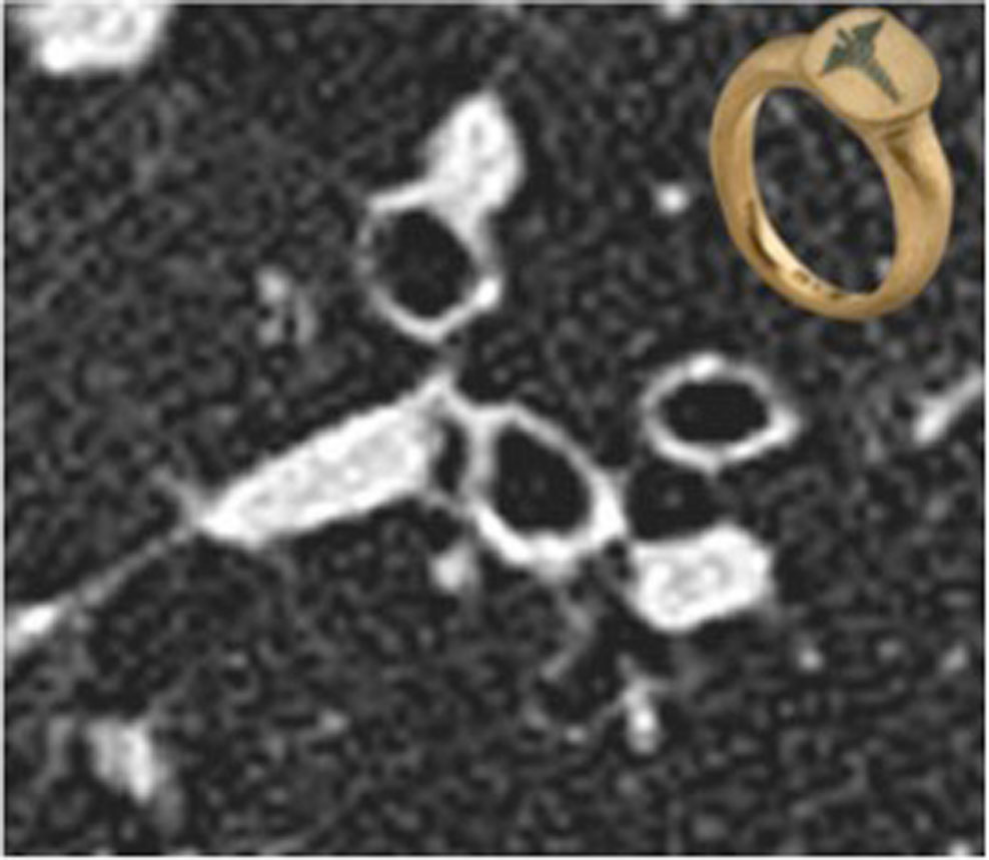
Axial high resolution CT image of dilated airways in a 19-year-old woman with cystic fibrosis. The airway diameter exceeds that of adjacent pulmonary artery resembling a jewelled or signet ring (inset)

The signet ring sign on chest CT refers to the appearance produced by a dilated bronchus, which exceeds the diameter of the adjacent pulmonary artery by a ratio of greater than 2:1. The dilated airway represents the hollow portion of the ring and the pulmonary artery represents the signet or jeweled portion (Fig. 21). Bronchiectasis is a result of bronchial wall damage leading to irreversible dilatation. It has many causes including infectious bronchitis, pulmonary fibrosis, cystic fibrosis (Fig. 21), and Kartagener syndrome [26].

## Veil-like opacity

**Fig. 22:**
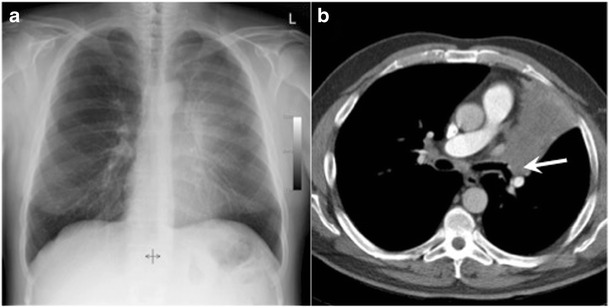
Chest radiograph (**a**) and axial CT image in a 42-year-old woman with pleuritic chest pain. A hazy opacity, similar to a veil, is projected over the left hemithorax (**a**) and confirmed to represent atelectasis due to an endobronchial lesion (arrow) at the origin of the left upper lobe bronchus on CT (**b**). Mild left hemidiaphragmatic elevation indicates volume loss (**a**)

Left upper lobe collapse can present as a radiographic veil-like opacity projected over the left hemithorax, this subtle opacity is a result of anterior collapse of the left upper lobe, which produces a subtle opacity rather than a sharp interface with aerated lung as the X-ray beam crosses the abnormality en face rather than tangentially (Fig. 22). Associated radiographic features include elevation of the left hilum and hemidiaphragm and a crescentic lucency between the mediastinum and the atelectatic upper lobe known as the Luftsichel sign. The crescentic lucency represents the upward displacement of the lingula [27].

## Conclusion

Certain pathologic conditions have classic radiologic manifestations that resemble clothing and accessories. These radiologic “clothing signs” help radiologists recall classic radiologic descriptions of pathologic appearances and narrow a differential diagnosis.
